# Role of Dendritic Cells in Exposing Latent HIV-1 for the Kill

**DOI:** 10.3390/v12010037

**Published:** 2019-12-28

**Authors:** Jan Kristoff, Charles R. Rinaldo, Robbie B. Mailliard

**Affiliations:** 1 Department of Infectious Diseases and Microbiology, Graduate School of Public Health, University of Pittsburgh, Pittsburgh, PA 15261, USA; jak83@pitt.edu (J.K.); rinaldo@pitt.edu (C.R.R.); 2 Department of Pathology, School of Medicine, University of Pittsburgh, Pittsburgh, PA 15261, USA

**Keywords:** dendritic cells, HIV-1 latency reversal, cytomegalovirus, T cells, CD40 ligand, immunotherapy, ‘kick and kill’

## Abstract

The development of effective yet nontoxic strategies to target the latent human immunodeficiency virus-1 (HIV-1) reservoir in antiretroviral therapy (ART)-suppressed individuals poses a critical barrier to a functional cure. The ‘kick and kill’ approach to HIV eradication entails proviral reactivation during ART, coupled with generation of cytotoxic T lymphocytes (CTLs) or other immune effectors equipped to eliminate exposed infected cells. Pharmacological latency reversal agents (LRAs) that have produced modest reductions in the latent reservoir ex vivo have not impacted levels of proviral DNA in HIV-infected individuals. An optimal cure strategy incorporates methods that facilitate sufficient antigen exposure on reactivated cells following the induction of proviral gene expression, as well as the elimination of infected targets by either polyfunctional HIV-specific CTLs or other immune-based strategies. Although conventional dendritic cells (DCs) have been used extensively for the purpose of inducing antigen-specific CTL responses in HIV-1 clinical trials, their immunotherapeutic potential as cellular LRAs has been largely ignored. In this review, we discuss the challenges associated with current HIV-1 eradication strategies, as well as the unharnessed potential of ex vivo-programmed DCs for both the ‘kick and kill’ of latent HIV-1.

## 1. Introduction

Despite major advances in human immunodeficiency virus-1 (HIV-1) treatment and prevention since the discovery of the virus in 1983 [1], the global rate of new infections remains constant at approximately 2 million per year [2,3]. Although optimal antiretroviral therapy (ART) suppresses viremia to below the limit of detection of clinical assays, HIV-1 is managed as a chronic disease due to its persistence in a long-lived population of resting memory CD4^+^ T cells considered to be the major reservoir [4,5]. Early treatment can limit reservoir size but not prevent its establishment, which occurs within days of initial infection [6]. Based on reservoir decay kinetics following ART initiation, it was initially predicted that 2–3 years of therapy would be sufficient to eliminate all HIV-infected cells [7]. However, rebound of viremia to pretreatment levels in HIV-infected individuals who discontinued therapy provided evidence for a longer-lived population of latently infected cells [8]. With a t_1/2_ of 44 months, this pool of infected cells would require approximately 73 years of therapy to eradicate [9]. Finding a safe and effective means to specifically expose this HIV-1 cellular reservoir for immune targeting and elimination during ART remains a major barrier to a functional cure. In this review, we dissect the potential strengths and limitations of current HIV cure strategies, with a particular emphasis on recent studies employing dendritic cell (DC)-based HIV-1 immunotherapies.

## 2. Dendritic Cell Programming for Immunotherapy

Derived from the bone marrow, DC serve as bridge between the innate and adaptive immune systems due to their dual capacity to respond to pathogen- and danger-associated signals during acute inflammatory responses and to process and present antigens for the priming of naïve T cells [10]. Although the principal human DC subsets in vivo consist of three main types including plasmacytoid DCs (pDCs) and myeloid/conventional DC1s (cDC1s) and DC2s (cDC2s) [10], models of DC ontogeny have undergone numerous revisions since the discovery of DCs by Ralph Steinman and Zanvil Cohn [11,12] more than 40 years ago. Due to recent experimental data supporting modification of the classical model of hematopoiesis that regulates DC lineage, it is now accepted that DCs originate not from a population of multipotent progenitor cells but from progenitors that follow predestined developmental pathways determined by lineage priming [13,14,15,16].

### 2.1. Classification of Endogenous DCs

DCs are further classified based on differential expression of transcription factors such as interferon regulatory factors 4 and 8, which distinguish plasmacytoid DCs (pDCs, IRF4^+^IRF8^+^), myeloid/conventional DC1s (cDC1s, IRF4^-^IRF8^+^), and myeloid/conventional DC2s (cDC2s, IRF4^+^IRF8^-^) [10,17,18,19,20]. Characterized by surface expression of CD123 and CD45RA, pDCs produce high levels of type I and type III interferons in response to viral infection in the forms of single-stranded RNA and double-stranded DNA detected by endosomal toll-like receptors 7 and 9, respectively [10,21,22].

Myeloid cDC1s were initially classified as a subset of blood DCs expressing high levels of CD141 (thrombomodulin) [23,24] but also reside in numerous lymphoid and nonlymphoid tissues [10]. Identification of cDC1s is also based on shared expression of CD13 and CD33 with cDC2s and low expression of CD11b, CD11c, and CD172 [10]; unique to cDC1s is the receptor that recognizes actin exposed during cell necrosis, CLEC9A [25,26]. Myeloid DC1s have a superior capacity to cross-present viral and intracellular antigens in the context of both MHC class I and class II for activation of CD8^+^ T cells and the induction of T helper type 1 (T_H_1) and natural killer (NK) cell responses [27,28,29,30]. Like pDCs, cDC1s produce both type I and type III interferons in response to pathogen sensing via TLR3 and TLR9 [10,31,32,33].

The predominant human cDC population in blood, as well as lymphoid and nonlymphoid tissues, cDC2s are characterized by various surface antigens, including CD1c, CD2, CD172, FcεR1, CD11b, CD11c, CD13, and CD33 [10]. Similar to monocytes, cDC2s express an extensive repertoire of lectins and pattern recognition receptors that facilitate immune responses to agents as diverse as intracellular pathogens, extracellular bacteria, parasites, and fungi [10]. Furthermore, cDC2s are capable of inducing T_H_1, T_H_2, T_H_17, and T_REG_ immune responses, due to their ability to secrete various pro- and anti-inflammatory mediators [10,34,35,36]. Of note, cDC2s are weak producers of type III interferons but can be stimulated to produce high levels of IL-12 compared to cDC1s [37,38,39,40].

### 2.2. Characterization of Ex Vivo-Programmed DCs

Ex vivo generation and programming of antigen-presenting, monocyte-derived DCs (MDCs) was first implemented in cancer immunotherapy to circumvent the dysfunction of endogenous DCs that occurs in cancer patients [41,42,43,44,45,46], capitalizing on the discovery of culture methods that were scalable to quantities required for clinical trials. In contrast to traditional vaccines that relied on antigen cross-presentation by patients’ own dysfunctional DCs, this strategy harnessed functional DCs generated outside of the tumor-associated suppressive environment to induce effective anti-cancer immunity [41,42,43,44,45,46]. This concept was based on the premise that MDCs could be imprinted during maturation with particular effector functions and homing properties for the induction of tumor-specific CD8^+^ T cells in vivo [47]. The first advantage of this approach is that ex vivo-generated DCs can be loaded with specific antigens that will promote effective delivery of both antigen (‘signal 1’) and costimulation (‘signal 2’) to antigen-specific T cells. However, the ability of DCs to cross-present antigen is affected by DC developmental stage and by the combination of factors used in their activation and maturation [48,49,50,51,52]. In addition, shortcomings of ‘first-generation’ DC vaccines highlighted the necessity for DCs to express high levels of costimulatory molecules and lymph node homing capacity in order to induce effective anti-cancer cytotoxic T lymphocyte (CTL) responses [47,53,54,55,56]. As a result, ‘second-generation’ DC maturation strategies incorporating PGE_2_ [57,58,59] were developed to induce fully mature DCs, with high expression of costimulatory molecules (CD83, CD86) and CCR7 (Table 1) [60,61,62,63,64] by activation via E-prostanoid receptors 2 (EP-2) and 4 (EP4) expressed on MDCs [65]. As a mediator of chronic inflammation, the addition of PGE_2_ mimics a microenvironment characteristic of a chronic inflammatory state such as that of a cancer setting. Although IL-1β/TNF-α/IL-6/PGE_2_-matured DCs displayed enhanced immunogenicity and migratory potential in response to lymph node-secreted chemokines CCL19 and CCL21 in healthy individuals [53,54,55,60,61], the negative impact of PGE_2_ on DC IL-12p70 production and their recruitment and activation of T_REG_ cells [66,67,68,69] was linked to limited anti-tumor [70] and anti-HIV clinical responses [71].

Since high IL-12p70 production by DCs has been shown to greatly enhance their ability to induce antigen-specific T_H_1 cells and CTL (‘signal 3’) [75,76,77,78,79,80,81,82,83,84,85], subsequent efforts to improve the efficacy of DC-based cancer vaccines have focused on employing ‘nonexhausted’ DCs, unlike the PGE_2_- matured DCs [59,62,86,87]. These ‘type 1-polarized’ monocyte-derived DCs (MDC1s) could be generated in the presence of type I and type II interferons and TLR ligands, or IL-18-activated NK cells or memory CD8^+^ T cells [62,67,72,88,89,90,91,92,93]. Both in vitro and in mouse models, high IL-12p70-producing DCs induced strong, long-lived tumor-specific CTL responses [47]. Furthermore, addition of IFN-γ and the combination of cytokine maturation cocktail reversed maturation-associated DC exhaustion, producing polarized DCs capable of increased IL-12p70 production in response to interaction with CD40L-expressing CD4^+^ T cells [62,67,92,94], mimicking an acute, pro-inflammatory microenvironment typically associated with successful anti-viral responses. Subsequent findings revealed that maturing MDCs enhanced expression of CCR7 and production of chemokines (‘signal 4’) that promote the interaction of MDC1 with naïve, memory, and effector T cells (Table 1) [69]. Specifically, inclusion of IFN-α and the TLR 3 ligand polyinosinic:polycytidylic acid [poly (I:C)] in the ‘alpha type 1-polarized’ DC (αDC1) maturation cocktail was found to augment production of CXCL9, CXCL10, CXCL11, and CCL5 to promote the interaction of mature DCs with CXCR3- and CCR5-expressing CTL, T_H_1, and NK cells desirable for effector responses [63,69,73,74]. Likewise, IFN-α-induced maturation results in reduced production of CCL22 by MDC1, thus avoiding attraction of undesirable CCR4-expresssing T_REG_ [69].

In sum, while differentially matured DCs can share similarities in antigen presentation, maturation status, and lymph node homing capacity, large discrepancies often exist in their capacities for optimal cytokine production, chemokine expression related to T cell trafficking, and antigen transfer (Table 1). Thus, correct ex vivo programming is critical to the design of DC-based immunotherapies in order to achieve the desired cellular mechanisms in vivo. Ex vivo generation of DCs allows manipulation of antigenic ‘signal 1’ and costimulatory ‘signal 2’ for optimal activation and expansion of both antigen-specific CD4^+^ and CD8^+^ T cells [47]. Education of DCs to further exploit polarizing ‘signal 3’ and homing ‘signal 4’ can selectively drive the desired antigen-specific T cell effector mechanisms and trafficking patterns. Together, such ex-vivo DC instruction could improve therapeutic outcomes by circumventing logistic issues related to the route of DC delivery, tissue-associated influences, cytokine exhaustion, and requirements for timely and functional interactions between DCs and T cells within lymphatic tissues [53,54,63,86,87,95,96,97,98].

## 3. Use of DCs to Address Hurdles to the CTL ‘Kill’ of HIV-1

Numerous clinical trials have shown that although HIV-1 proviral reactivation is achievable in vivo, the elimination of reactivated cells is minimal to nonexistent [99,100,101,102,103,104,105]. These results highlight that the ‘kill’ does not necessarily follow latency reversal, regardless of sufficient expression of viral RNA or antigens by infected cells [106,107]. CD8 depletion studies in SIV-infected nonhuman primates support the crucial role of CD8^+^ T cells in control of viral replication [108,109,110,111,112]. Likewise, human studies have found temporal associations between a peak in HIV-specific CD8^+^ T cell responses during acute infection, control of HIV viremia, and delayed disease progression [113,114,115]. A successful anti-HIV-1 response likely requires either the reactivation of memory CTL or the activation of de novo CTL responders capable of effective recognition of cells expressing peptide antigen derived from replication-competent proviruses following latency reversal [116]. While DC-based strategies may offer a route to educate and assemble an arsenal of CTLs, the challenges associated with effective targeting of latently infected cells are multifactorial.

### 3.1. Mutations in CTL Epitopes

CTL recognition of HIV-1 epitopes is hampered by escape mutations that are selected early in infection due to heightened immune pressure [117,118,119,120] and continue to contribute to the latent reservoir until the initiation of ART [116]. Proviruses with escape mutations to immunodominant CTL epitopes comprise a significant portion of the latent reservoir in individuals who initiate ART in the chronic stage of HIV-1 infection [121]. In addition to the establishment of CTL escape variants, minor viral modifications to CTL epitopes can also occur, leading to a partial or incomplete immune escape resulting in the induction of dysfunctional cross-reactive memory responses characterized by the production of proinflammatory cytokines by responding CTLs in the absence of target cell killing [74,122]. In a sense, this can create type of immunologic smokescreen, which could be even more problematic than the establishment of a complete CTL escape variant, since the responding effector cells can act as competitive inhibitors for the induction and effector activity of new antigen-specific CD8^+^ T cells. Such dysfunctional cross-reactive CTLs could negatively impact the afferent phase of the immune response by competing for antigen presented by DCs, potentially affecting their capacity to generate de novo killer CTL responses against the newly established variants, as well as to block the ability of newly activated CTLs to target the infected cells in the periphery during the efferent phase of the immune response. Moreover, cytokines produced by cross-reactive CTLs targeting these adaptive epitopes can create an environment to promote chronic inflammation and DC-mediated HIV-1 dissemination [74,123]. Using MDC1 to selectively prime HIV-1-specific CD8^+^ T cells from naïve T cell precursors while avoiding pre-existing, dysfunctional memory T cells has been shown effective in vitro for targeting persistent HIV in individuals on ART [122].

Thus, successful CTL-based HIV eradication strategies should target subdominant viral epitopes or epitopes for which escape mutations have not accumulated [121]. Graph-based epitope selection strategies using mathematical algorithm program tools such as Epigraph can be used to assess global epitope diversity and/or conservation when designing DC vaccine antigenic formulations [124]. An alternative to this approach is to base antigen selection according to biochemical network analyses of CD8^+^ T cell epitope topology based on HIV proteome data, which has revealed an inverse relationship between protective CTL epitopes targeted by HIV controllers and mutational frequency in vivo [125]. An epitope that is determined to have a high network value indicates that it is contained within an important structural region of the viral proteome, critical for maintaining the three-dimensional protein structure and function, and thus important to the overall fitness of the virus. Utilizing DCs to selectively re-focus the CTL response away from highly variable antigenic targets and towards these critical regions of the virus is an attractive notion.

### 3.2. Dysfunctional or Exhausted CTLs

CTL exhaustion is another critical consideration in the design of effective kill-phase strategies. T cell exhaustion can occur in the settings of chronic viral infections or cancer, as a result of persistent immune activation and antigen exposure [116,126,127,128,129]. Among defects in CTL function that arise during chronic HIV-1 infection, exhaustion cannot be fully reversed with ART [130]. Exhaustion in antigen-specific T cells involves a gradual loss of effector function, accompanied by decreased proliferative capacity [131]. Compared to terminally differentiated or memory CD8^+^ T cells, exhausted CD8^+^ T cells are characterized by decreased cytokine production and cell surface expression of inhibitory receptors including PD-1, TIM-3, CTLA4, CD160, LAG-3, and TIGIT [131,132,133,134,135,136]. Increases in inhibitory receptor expression serve as immune checkpoints (ICs) to limit excess T cell activation during a normal immune response and are downregulated upon its resolution [134,137]. Conversely, sustained expression of inhibitory receptors results from persistent antigen exposure, leading to an exhausted phenotype. Even in the context of suppressive ART, in which exposure to HIV antigen is limited, it may not be possible to completely restore CTL functionality [116]. This is illustrated by high levels of PD-1 and TIGIT expression by both follicular and nonfollicular CD8^+^ T cells in the lymph nodes of HIV-infected individuals [138]. However, immune checkpoint blockade combination therapies approved for use in cancer immunotherapy have been shown to reverse exhaustion to varying degrees in virus-specific T cells [128,136,139,140]. In vitro blockade of the PD-1/PD-L1 axis in follicular and nonfollicular lymph node cells was shown to restore HIV-1-specific CD8^+^ T cell function [138], and a phase I clinical trial of the anti-PD-L1 antibody BMS-936559 demonstrated a trend toward enhanced Gag-specific CD8^+^ T cell responses in virally suppressed participants [141] Importantly, correlations between HIV DNA content in memory CD4^+^ T cells and expression of PD-1, TIGIT, and LAG-3 inhibitory receptors have been documented, suggesting a potential role for these ICs in latency establishment [116,142,143].

### 3.3. Defective HIV as Antigen Decoys

Another challenge of the ‘kill’ phase is the excess of defective proviruses that occupy the latent reservoir in vivo. These defective proviruses (HIV-1_def_) can result from hypermutations, large internal deletions, packaging signal deletions, major splice donor (MSD) mutations, or inactivating point mutations [144,145,146], and significantly outnumber cells harboring replication-competent proviruses. As a result of intact LTR promoter function, integration into actively transcribed genes, and lack of promoter methylation, some HIV-1_def_ can be transcribed, translated, and recognized by HIV-specific CTLs [145,146,147], thus serving as decoy targets that act to divert the CTL-mediated elimination of the true latent reservoir [145,148]. It has been postulated that preferential targeting of HIV-1_def_ may be a function of their ability to present antigen to CTLs more efficiently as a result of a defect in Nef and the inability to downregulate MHC class I [145]. Evidence that clonal expansion of cells containing both defective and replication-competent HIV-1 shapes the pool of latently infected cells in individuals on long-term ART [149,150,151,152,153] poses an additional challenge to targeting only those proviruses.

### 3.4. Spatial Separation of CTL and Target Cells

One of the greatest challenges of targeting the HIV reservoir for CTL-mediated elimination is the spatial separation of target and effector cells in anatomical ‘sanctuaries’ [154] that serve as a persistent source of latent provirus. For example, the CD4^+^ T follicular helper (T_FH_) and T follicular regulatory (T_FR_) cell subsets within B cell follicles of lymph nodes are more permissive to HIV-1 infection than extrafollicular subsets ex vivo [155,156,157] and comprise a major portion of the latent reservoir during both chronic SIV and HIV infection [158,159,160,161]. Whereas CXCR5^+^ CD8^+^ T cells have been identified within the B cell follicles of secondary lymphoid organs during chronic viral infections [162,163,164], CTLs could be excluded from the B cell follicle because they usually lack the follicular homing receptor CXCR5, which is not expressed until late in untreated infection [158]. As a result, target cells within the follicle are protected from CTL-mediated cytolysis, whereas viral replication in extrafollicular zones is efficiently controlled [165]. Indeed, ongoing SIV replication within T_FH_ cells of elite controller macaques has been attributed to CTL exclusion from B cell follicles, as depletion of SIV-specific CD8^+^ T cells resulted in increased viral replication in non-T_FH_ CD4^+^ T cells [154,166].

Mechanisms governing CTL migration to and cytolytic activity within B cell follicles are poorly understood, and novel strategies to address barriers to CTL targeting of this compartment are being explored. Among these, temporary disruption of the B cell follicle with depleting antibodies (anti-CD20/rituximab), blockade of T and B cell interactions using anti-CD40L, and therapeutic vaccination with engineered CTL or chimeric antigen receptor T cells expressing CXCR5 have been proposed [154,165]. Transduction of CTLs with CXCR5 to facilitate B cell follicle homing [167] and in vitro stimulation with TGF-β to induce CXCR5 expression in CXCR5^-^CD8^+^ T cells has been demonstrated in rhesus macaques [168]. Another promising approach involves the use of bispecific antibodies targeting CD3 and HIV gp120 that have the dual capacity to act as latency reversal agents (LRAs), through a CD3-mediated activation of CD4^+^ T cells containing HIV provirus, and to facilitate CD8^+^ T cell-mediated ADCC of the reactivated HIV-infected CD4^+^ T cells induced to express Env protein. [169]. Although this remains to be tested in vivo, lymph node-homing CXCR5^+^CD8^+^ T cells could be transduced to produce these antibodies in order to facilitate killing of latently infected cells within the B cell follicle [165]. A better understanding of the signals required to drive CXCR5 expression in CTLs during priming could lead to the optimal design of ex-vivo-educated DCs purposely programmed to provide such needed factors.

## 4. Driving HIV-1 out of Hiding: Current Status of Latency Reversal Approaches

Beyond spatial separation of target and effector cells, the greatest obstacle to the ‘kill’ is HIV-1 latency itself. The ‘kick and kill’ approach to HIV eradication entails proviral reactivation during ART, coupled with generation of CTLs or other immune effectors equipped to eliminate exposed infected cells [170]. Effective yet nontoxic strategies to target the latent reservoir remain elusive, due to limited post-integration proviral gene expression that spares infected cells from viral cytopathic effects or effector-mediated clearance [171].

### 4.1. Common LRA Strategies

Perhaps the largest class of LRAs in current use, epigenetic modifiers, include histone deacetylase inhibitors (HDACi), histone methyltransferase inhibitors (HMTi), DNA methyltransferase inhibitors (DNMTi), bromodomain and extraterminal (BET) bromodomain inhibitors (BETi), positive transcription elongation factor b (P-TEFb) activators, and hexamethylene bisacetamide (HMBA) [172,173]. All of these compounds operate at various levels of transcriptional control to mediate changes in chromatin structure that permit proviral reactivation. Among these, HDACi have been most extensively studied, and clinical trials of vorinostat, panobinostat, and romidepsin have all demonstrated HIV reactivation in vivo [99,102,104,105,174]. However, none of these agents impacted levels of HIV-1 DNA.

PKC agonists have been shown to induce HIV LR through activation of NF-κB signaling by intracellular PKC isoforms [173]. Within this class of LRAs, the phorbol esters prostratin and 12-deoxyphorbol 13-phenylacetate (DPP) have both demonstrated potential as T cell activators and LRAs in latently infected primary cells ex vivo [175,176]. In addition, the PKC agonist bryostsin-1 has been used to achieve potent T cell stimulation both in vitro and ex vivo [177,178]. However, its association with adverse effects in clinical oncology trials [179,180,181,182] prompted conservative drug dosing of bryostatin-1 in a subsequent HIV-1 clinical trial, resulting in neither PKC activation nor HIV reactivation [103].

Importantly, both ex vivo studies and clinical trials have demonstrated that no single pharmacological LRA in current use has been able to reactivate a significant proportion of the latent HIV-1 reservoir [183,184]; therefore, combination LRA approaches are being explored [173]. An additional complication of proposed pharmacological LRAs is that many demonstrate the capacity to negatively impact CTL and NK cell effector function [178,185,186,187].

### 4.2. Next Generation Pharmacological LRAs?

Among LRAs that have shown the most promise, TLR agonists have been investigated in several ex vivo and in vivo studies [188,189,190]. The TLR7 agonist GS-9620 (Vesatolimod) in combination with the HIV envelope-specific broadly neutralizing antibody (bNAb) PGT121 induced extracellular HIV-1 RNA and increased cytolytic activity in PBMCs of virally suppressed individuals [188]. Though the mechanism of HIV reactivation in this study has not been fully elucidated, both latency reversal and T cell activation were dependent on type I IFNs produced through TLR7 stimulation of pDCs. Co-administration of PGT121 and Vesatolimod during ART also delayed viral rebound in SHIV-infected rhesus macaques upon ART discontinuation [190]. Finally, combined Ad26/MVA therapeutic vaccination/TLR7 stimulation was shown to increase the breadth of SIV-specific CTL responses, decrease levels of SIV DNA, and delay viral rebound following ART interruption in SIV-infected rhesus macaques [189]. Vesatolimod has since advanced to human trials in both ART-suppressed participants (NCT02858401) and HIV-1-infected controllers on ART and during ATI (NCT03060447).

The efficacy of TLR9 agonist MGN1703 as an innate immunity enhancer and LRA was evaluated in HIV-1-infected, ART-suppressed individuals (http://www.clinicaltrials.gov NCT02443935) [191]. Whereas activation of pDCs, increases in plasma IFN-2α and HIV-1 RNA, upregulation of ISG transcription, and increased proportions of activated NK and CD8^+^ T cells resulted from MGN1703 treatment, dosing did not impact reservoir size [191]. The TLR9 agonist lefitolimod in combination with bNAbs is currently being evaluated in HIV-1-infected individuals on ART and during ATI for its impact on HIV-1 reservoir reduction (NCT03837756).

Finally, in addition to its ability to enhance HIV-specific CTL responses [165,192], IL-15 is being investigated for its LRA potential as a novel LRA. IL-15 is a product of activated DCs, which stimulates antiviral NK and CD8^+^ T cell responses [193,194,195] when presented in *trans* as part of a membrane-bound IL-15:IL-15Rα complex [194,196]. IL-15 superagonists recapitulating this biologically potent heterodimer functionality are being explored as potential LRAs [192]. Both IL-15 and the IL-15 superagonist ALT-803 induced LR activity in a primary CD4^+^ T cell model of HIV latency, and ALT-803 also enhanced CTL killing of HIV-infected cells ex vivo. In addition to being evaluated in human cancer trials (NCT01946789, NCT01885897, NCT02099539), dose escalation studies of ALT-803 are being performed to assess whether it can be tolerated at doses deemed safe in nonhuman primates.

## 5. Dual Role for DCs in the ‘Kick and Kill’?

### 5.1. DCs as a Therapeutic Tool to Drive HIV-1-Specific Killer T cells

A revolutionary study by Lu et al. in SIV-infected rhesus macaques revealed the promise of therapeutic dendritic cell vaccination using inactivated SIV-loaded autologous DCs [197]. Three immunizations elicited a 50-fold decrease in SIV DNA and a 1000-fold decrease in SIV RNA in peripheral blood that were sustained throughout the study and correlated with increased SIV-specific cellular and humoral responses. These impressive results were replicated in a subsequent trial in chronically HIV-infected, untreated individuals who exhibited prolonged post-vaccination suppression of viral load that was attributed to strong virus-specific CD4^+^ T helper and CD8^+^ effector responses [198].

An early DC-based HIV immunization strategy developed by our group implemented autologous mature DCs pulsed with HLA*A02-restricted HIV-1 Gag, Pol, and Env peptides and influenza A matrix protein peptide administered to participants intravenously or subcutaneously [199]. Although the peptide-DC vaccine elicited HIV-specific IFN-γ responses at two weeks following the second immunization, the DCs used were suboptimal for the induction of long-lived, broadly reactive CTL responses. However, one of the most impressive HIV immunotherapy trials to date utilized DCs pulsed with inactivated autologous HIV, which resulted in a 1 log_10_ decrease in HIV RNA setpoint and was associated with increased anti-HIV CD8^+^ T cell IFN-γ responses [200]. Nonetheless, as with many of these earlier DC-based studies, this trial implemented DC generation methods that yield IL-12p70-deficient DCs incapable of inducing sustained HIV-specific effector responses. In an attempt to address this issue, Argos Therapeutics investigated ex vivo genetic manipulation of DCs as a strategy to deliver a constitutive CD40L helper signal to the DCs in an HIV immunotherapy to treat acute and chronic infections [201,202,203]. Autologous monocyte-derived DCs were co-electroporated with synthetic CD40L RNA and HIV RNA encoding Gag, Nef, Vpr, and Rev derived from individuals’ pre-ART plasma to create the personalized AGS-004 vaccine [204]. Nevertheless, this approach was unsuccessful, which may have been due to the fact that constitutive CD40L signaling induces an early burst of IL-12p70 production, but ultimately creates IL-12p70-exhausted DCs that are unresponsive to CD4^+^ T_H_ cell interaction [122].

A novel therapy proposed by Guardo et al. combined TRIMIX adjuvant and an HIV T cell immunogen (HTI) for in vivo targeting of DCs by intranodal injections [205]. The previously described TRIMIX adjuvant consists of three mRNAs encoding CD40L, the costimulatory molecule CD70, and constitutively activated TLR4 [206]. The HTI vaccine component consists of an mRNA expressing epitopes of Gag, Pol, Vif, and Nef proteins, chosen on the basis of antigen-specific CD4^+^ and CD8^+^ T cell reactivity [207]. Monocyte-derived DCs electroporated with this preparation were shown to induce T cell proliferation and IFN-γ responses in vitro, and intranodal injection of TRIMIX/HTI induced antigen-specific CTL responses in mice [205]. In addition, human lymph node explants treated with TRIMIX/HTI activated DCs and induced proinflammatory mediator production. However, the IL-12-producing capacity of the mRNA/DC-based formulation was not investigated in this study, therefore providing no information regarding its potential to induce broadly reactive CTLs required for the long-term control of viremia in the absence of ART [208].

More recently, Surenaud et al. reported increased HIV-specific CD8^+^ and CD4^+^ T cell responses in patients on ART following therapeutic vaccination with DCs generated ex vivo in the presence of IFN-α and loaded with a combination of Gag, Pol, and Nef peptides (LIPO-5) [209]. HIV-specific CD8^+^ and CD4^+^ T cells targeted both dominant and subdominant epitopes of Gag, Pol, and Nef and elicited IL-2 and IL-13 production by CD4^+^ T cells that significantly correlated with control of viremia upon analytic treatment interruption (ATI). In addition to predicted epitopes, the vaccine also elicited CD4^+^ T cell responses against previously unidentified HIV-1 HLA-DR-restricted CD4^+^ T cell epitopes, highlighting the importance of CD4^+^ T cell responses in control of HIV-1 viremia.

Overall, although DC-based HIV-1 immunotherapies have proven to be safe and well tolerated, they have achieved a success rate of only 38% according to meta-analyses [210]. Furthermore, while there have been many DC-based therapeutic approaches explored in HIV clinical trials, there has been little consensus with regards to the selection of subjects, methods of DC generation and preparation, choice of immunogen, and assessment of immunologic and virologic responses [211]. These parameters will be critical to the design of future trials, and although beyond the scope of this review, efforts are being made to compare and interpret outcomes of DC-based HIV vaccination strategies to date.

### 5.2. LRA Potential of DCs

Although DCs have been used extensively for the induction of antigen-specific T cell responses in HIV-1 clinical trials [199,200,212,213], their capacity to function as an LRA has only gained recent attention. In fact, as DCs are intended to engage in cognate interaction with CD4^+^ T cells, it is not surprising that they have been implicated in both HIV *trans* infection [214,215] and latency establishment [216,217]. For the same reason, it is plausible that DCs could be utilized to reactivate provirus from these same cells. Interestingly, results of a recent phase I/II clinical trial developed by our group linked the administration of DCs loaded with autologous HIV that were designed to induce CTL responses [218] with post-vaccination increases in plasma viremia in ART-suppressed participants [219]. As the aim of that study was not to evaluate the efficacy of the DC therapeutic as an LRA, pertinent questions regarding the underlying mechanisms of the observed proviral reactivation remain.

Historically, most DC-based strategies aimed at the ‘kick’ of latent HIV in vitro have employed monocyte-derived immature DCs (iDCs) and DCs matured by exposure to bacterial antigens [220,221,222]. Among these studies, several have purported that DC-mediated LR could be induced by DC–T cell contact in the absence of antigen presentation and/or mediated by DC-secreted soluble factors. For example, van der Sluis et al. reported that the interaction of MDC with actively proliferating primary T cells resulted in secretion of unidentified components by DCs to induce latent provirus [220]. However, in this study, iDCs were cultured with T cells that had been preactivated using either PHA or anti-CD3/CD28 beads. These treatments alone have been shown to reverse HIV-1 latency [184,223,224,225,226], and thus, it is difficult to dissect the clinical relevance.

In another study, CD40L-transduced DCs were reported to induce latency reversal in J-Lat and ACH-2 cell lines, independent of DC–T cell contact and mediated by TNF-α [227]. Similarly, MDCs matured by microbial components related to AIDS-associated pathogens, including *Mycobacterium bovis*, *Bacillus Calmette-Guérin* (BCG), and lipopolysaccharide (LPS), were shown to reactivate latent HIV in Jurkat T cells through secretion of TNF-α [222]. Although the incorporation of antigen in DC-mediated LR has been underexplored, Marini et al. successfully utilized iDCs loaded with SEB superantigen for reactivation of HIV-1 in in vitro–infected CD4^+^ T cell lines [221]. Furthermore, van Montfort and colleagues determined that iDCs in combination with TCR stimulation more efficiently reversed latency in primary HIV-infected cells from aviremic individuals than TCR activation alone, a phenomenon that was dependent on PI3K-Akt-mTOR pathway activation [228]. Finally, a study investigating the latency activation potential of various DC subsets demonstrated that tissue-resident and blood-derived myeloid DCs reactivated virus in vitro from latently infected effector T cells with different efficiencies [229]. Nevertheless, none of these DCs therapeutics was designed with the dual purpose of creating sufficient HIV-1 antigen exposure for successful CTL targeting of the CD4^+^ T cell reservoir. Information regarding the antigen specificity of CD4^+^ T cells harboring HIV would be an important step in designing a DC-based therapy to directly activate viral production from these cells.

### 5.3. Using DCs to Expose Pathogen-Specific HIV Reservoirs?

Previous studies have shown that HIV-1 preferentially infects and depletes HIV-specific CD4^+^ T cells of HIV infected individuals [230,231], but some of these latently infected cells survive and retain their antigen specificity during ART [232]. Therefore, HIV-1 specific CD4^+^ T cells may themselves harbor a portion of the long-lived latent reservoir and can potentially be exposed upon their antigen-specific activation. This could partially explain why our early HIV-1 antigen-loaded DC-based therapy was found to subsequently drive residual viremia in ART-suppressed individuals following ATI [219].

Despite that it has been reported that CMV-specific CD4^+^ T cells are less susceptible to HIV-1 infection in vivo [233], they were still found to be susceptible to a degree. Moreover, susceptibility to infection may not be directly related to the capacity of these cells to survive and clonally expand to harbor persistent infection over time. In fact, a large body of data exists to support the notion that there is a sizeable contribution of CMV antigen-specific CD4^+^ T cells to the latent reservoir. For example, the seroprevalence of CMV among HIV-infected individuals is extremely high, with conservative estimates being greater than 95% [234], and levels of CMV-specific CD4^+^ T cells as great as 25% have been documented in those who are coinfected [235,236,237,238]. In the mucosal and peripheral tissues of HIV-1-infected individuals, CMV hijacks human cytokine/chemokine signaling to fuel inflammation, thereby augmenting its own replication [239]. Subclinical CMV replication not only creates an environment conducive to reservoir seeding by recruiting target cells to sites of inflammation that may be shared sites of HIV persistence [240,241,242,243], but ultimately contributes to T cell dysfunction, impaired immune cell recovery, and chronic immune activation during ART [244,245,246,247]. Interestingly, Agudelo-Hernandez et al. [248] found a negative correlation between higher rates of subclinical herpes virus shedding and levels of inflammation and immune activation in virally suppressed, HIV-infected men. Nevertheless, CMV replication in the gut of ART-suppressed individuals has been associated with inflammation, mucosal barrier damage, and microbial translocation [240]. Finally, upregulation of CCR5 expression on susceptible target cells in the shared inflammatory environment triggered by CMV [249] and its ability to manipulate AP-1 and NF-κB signaling for the induction of HIV-1 gene expression [250,251,252,253] also serve to amplify HIV infection in CMV-coinfected individuals.

If established within CMV-specific CD4^+^ T cells, the latent HIV-1 reservoir could undergo cellular proliferation or clonal expansion in response to chronic CMV antigenic stimulation [242]. Data from several studies corroborate this theory. For example, a cross-sectional study of ART-naïve and ART-suppressed participants revealed a correlation between elevated levels of HIV-1 DNA and CMV replication in semen and peripheral blood [254,255]; another associated delayed decay of HIV DNA reservoirs with CMV replication in PBMC of men initiating ART during acute infection [243]. In addition, proviral and integration site analyses point to clonal expansion of latently infected cells as a major driver of HIV persistence under suppressive ART [149,150,153,256,257]. Evidence of selective increases in HIV DNA within CMV- and EBV-specific CD4^+^ T cells after immune reconstitution in HIV-infected patients who received chemotherapy for CMV- and EBV-associated malignancies illustrates this phenomenon [258]. Finally, self-renewal of stem cell memory T cells (T_SCM_), which were reported to contain the most copies of integrated provirus per cell in HIV-1-infected individuals, has been implicated to play a role in expansion of the latent HIV reservoir [259]. Of note, CMV-/HIV-coinfected persons possess functional CMV-specific T_SCM_ that may undergo homeostatic proliferation during ART to promote reservoir expansion [260]. Coincidentally, expanded CMV-specific CD4^+^ T cells harboring latent HIV could be protected from elimination as a consequence of numerous immune evasion strategies that CMV has evolved, including production of decoy viral homologues of HLA class I molecules [261,262] and cytokines [263,264], hijacking of cell signaling pathways [142,263,264,265], and inhibition of apoptosis [266,267]. These findings suggest that CMV coinfection contributes to establishment and maintenance of the HIV-1 reservoir, and that CMV antigen-loaded DCs could offer a means to selectively expose a portion of the HIV-1 reservoir contained within CMV specific CD4^+^ T cells.

### 5.4. DC Potential as an All-in-One ‘Kick and Kill’ Tool

**Figure 1:**
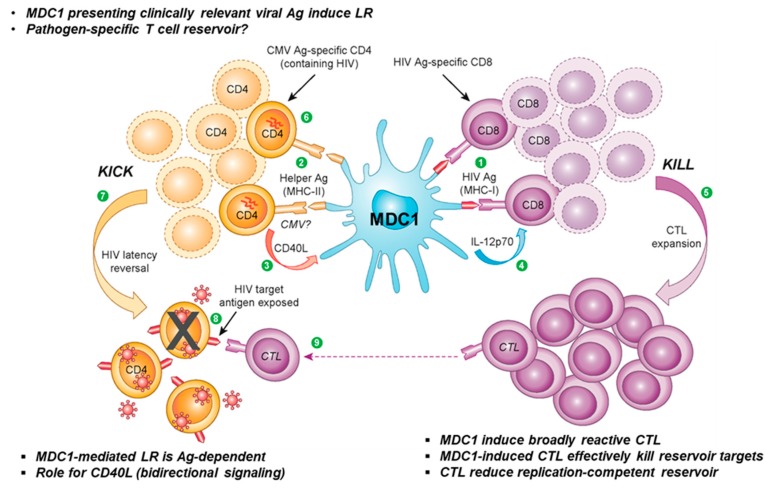
Monocyte-derived DC (MDC1): the all-in-one ‘kick and kill’ tool. MDC1s induce antigen-specific CD8^+^ and CD4^+^ T cell responses through presentation of antigenic peptides in the context of **(1)** MHC class-I and **(2)** MHC class-II molecules, respectively (signal 1), along with costimulatory factors including CD80 and CD86 (not shown, signal 2). Responding CD4^+^ T cells subsequently provide MDC1 with the feedback hyperactivating ‘helper’ signal CD40L **(3)**, necessary for MDC1 release of IL-12p70 **(4)**, which then promotes expansion and differentiation of CD8^+^ human immunodeficiency virus-1 (HIV-1)-specific effector cytotoxic T lymphocytes (CTLs) **(5)**. Activation of CMV and HIV antigen-responsive CD4^+^ T cells harboring latent HIV-1 **(6)** results in HIV latency reversal **(7)**, with HIV-1 proteins being transcribed and expressed as surface antigen **(8)**. As a result, exposed infected cells harboring replication-competent provirus are targeted for elimination by HIV-specific CTLs **(9)**.

In a recent study from our group, we demonstrated the ability of a clinically applicable DC-based therapeutic strategy to facilitate both the ‘kick’ and ‘kill’ of latent HIV-1 in CD4^+^ T cells from chronically infected, ART-suppressed individuals (Figure 1). Antigen-presenting type 1-polarized DC (MDC1) induced the transcription of replication-competent provirus, as well as HIV-1-specific CTL responses that targeted exposed infected cells for immune elimination [268]. The induction of strong antigen-specific CTL responses is attributed to uniquely high IL-12 production by MDC1 in response to interaction with CD40L-expressing CD4^+^ T helper cells [64]. Such MDC1s have already been successfully implemented in clinical oncology trials [47,80,269,270,271,272] due to their superior ability to drive long-lived CTL responses from naive T cell precursors [273,274] and have been approved for use in phase I HIV clinical trials. Likewise, strategic loading of MDC1 with MHC class II viral antigens (CMV, HIV-1) to facilitate interaction with CD4^+^ T_H_ cells reactivated latent provirus. Thus, this dual therapeutic approach could prove a safer, more targeted strategy for purging the latent HIV-1 cellular reservoir.

Notably, there were some caveats and limitations to our study. Although the underlying mechanisms of MDC1-mediated latency reversal have not been fully elucidated, bidirectional cross-talk between MDC1 and CD4^+^ T cells during cognate antigen-driven interaction involving CD40/CD40L signaling plays a role in the observed LR activity [268]. Furthermore, while antigen-driven, we cannot exclude the possibility that the MDC1-mediated proviral reactivation demonstrated represents nonspecific bystander effects of a potent antigen-specific response, instead of a direct impact on an antigen-specific HIV reservoir. As such, these questions warrant further investigation.

## 6. Concluding Remarks

While current data from our group and others indicate that the pool of both CMV- and HIV-1-specific CD4^+^ T cells contributes to the latent HIV-1 reservoir, it is probable that CD4^+^ T cells specific to other viruses that manifest as chronic infections, especially EBV and other herpes viruses, also harbor replication-competent provirus. It is well documented that secondary lymphoid organs such as the spleen, lymph nodes, and GALT are the major sites of HIV replication and persistence, and targeting the true latent reservoir within B cell follicles of these anatomical sanctuaries poses numerous challenges with regard to antigen delivery by a DC vaccine in vivo [160]. Furthermore, studies in nonhuman primates have clearly demonstrated that viral RNA may be found in a diverse array of organ systems not limited to lymphoid tissues, including the brain, kidneys, liver, and lungs [275,276,277]. As previously detailed, novel strategies to address immune targeting of these compartments are being developed [138,154,165,169,192]. As such, the LRA potential of other antigen-presenting cells such as B cells could be considered as strategy for targeting T_FH_ within lymph node follicles. Additionally, to broadly target other anatomical sites, exploring the use of DC-derived exosomes might prove useful, since they may be able to widely circulate and present DC-associated proteins and/or antigens to T cells throughout the body, and have been shown to have T cell activating capacity [278]. Alternatively, developing methods for in vivo antigen delivery to resident antigen-presenting cells in these compartments should be pursued. Nevertheless, successful cure strategies will employ nontoxic, directed inducers of latency reversal, coupled with methods to augment immune effector functions. Mounting evidence suggests that this goal will entail identification of the antigen specificities of latent cellular reservoirs and may be best achieved through incorporation of antigen-presenting ex vivo-educated DCs.

## Figures and Tables

**Table 1:** Immunotherapeutic potential of ex vivo-educated dendritic cells (DCs).

Desired Trait	PGE_2_ DC	Type 1-polarized DC	References
Antigen Presentation	++++	++++++	[52]
Maturation status (high CD83 / CD86)	++++++	++++++	[62,64]
Lymph node homing (CCR7)	++++++	++++	[60,61,62,63,64]
IL-12p70, IL-15 production	Deficient	++++++++	[62,64,72]
T cell trafficking / chemokine expression	CCL22 (T_REG_)	CCL19 (T_N_, T_CM_)	[63,69,73,74]
		CCL3-5, CXCL9-11 (T_EM_)	
Antigen transfer ability	Deficient	++++++	[64]
